# Accepting Lower Salaries for Meaningful Work

**DOI:** 10.3389/fpsyg.2017.01649

**Published:** 2017-09-29

**Authors:** Jing Hu, Jacob B. Hirsh

**Affiliations:** ^1^Rotman School of Management, University of Toronto, Toronto, ON, Canada; ^2^Institute for Management & Innovation, University of Toronto Mississauga, Mississauga, ON, Canada

**Keywords:** meaning, purpose in life, money, compensation, organizational behavior, meaningful work

## Abstract

A growing literature indicates that people are increasingly motivated to experience a sense of meaning in their work lives. Little is known, however, about how perceptions of work meaningfulness influence job choice decisions. Although much of the research on job choice has focused on the importance of financial compensation, the subjective meanings attached to a job should also play a role. The current set of studies explored the hypothesis that people are willing to accept lower salaries for more meaningful work. In Study 1, participants reported lower minimum acceptable salaries when comparing jobs that they considered to be personally meaningful with those that they considered to be meaningless. In Study 2, an experimental enhancement of a job’s apparent meaningfulness lowered the minimum acceptable salary that participants required for the position. In two large-scale cross-national samples of full-time employees in 2005 and 2015, Study 3 found that participants who experienced more meaningful work lives were more likely to turn down higher-paying job offers elsewhere. The strength of this effect also increased significantly over this time period. Study 4 replicated these findings in an online sample, such that participants who reported having more meaningful work were less willing to leave their current jobs and organizations for higher paying opportunities. These patterns of results remained significant when controlling for demographic factors and differences in job characteristics.

## Introduction

Work plays an important role in human life, with most working-age adults spending a large portion of their waking hours in work settings. Given the centrality of work, it is perhaps no surprise that workplace experiences can strongly influence a person’s well-being (Greenhaus et al., 1987; Tait et al., 1989; Judge and Watanabe, 1993). On the one hand, work can provide a sense of economic security through financial reward. In addition to satisfying these monetary concerns, however, work can also provide an individual with a sense of purpose, meaning, and identity (Ryan and Deci, 2001; Pratt and Ashforth, 2003; Rosso et al., 2010). The desire to experience a sense of meaning in one’s actions has in fact been theorized as one of the primary sources of work motivation (Hackman and Oldham, 1980; Barrick et al., 2013). People who are able to derive a sense of meaning from their work enjoy many benefits, including enhanced motivation, productivity, and well-being (May et al., 2004; Steger et al., 2012). In contrast, a lack of meaningful work has long been recognized as a primary source of alienation, anxiety, emotional exhaustion, and boredom in the modern era (Seeman, 1959; Kanungo, 1982; Maslach et al., 2001; Shantz et al., 2014).

Despite the accumulated evidence that work can provide a sense of meaning in addition to financial rewards, little research has directly gauged how these two aspects of work relate to each other. Some research suggests that perceiving one’s work as highly meaningful can lead to various forms of self-sacrifice, including the acceptance of unpaid or underpaid labor in the name of one’s calling (Bunderson and Thompson, 2009; Dempsey and Sanders, 2010). To expand our understanding of these relationships, the current paper explores whether the experience of work meaningfulness can influence the importance of financial compensation when making job choices. Specifically, the goal of the present set of studies is to examine whether, and to what extent, people are willing to accept lower salaries for more meaningful work opportunities.

Work meaningfulness has been defined as the “degree to which an employee experiences the job as one which is generally meaningful, valuable, and worthwhile” (Hackman and Oldham, 1976, p. 162). The experience of meaningful work is thus rooted in an individual’s subjective judgment of the work’s personal and social significance, with more meaningful jobs providing a deeper sense of purpose and value (Pratt and Ashforth, 2003; Grant, 2008; Rosso et al., 2010; Schnell et al., 2013).

Experiencing one’s life as meaningful has been identified as a central component of human well-being (Ryff, 1989; Ryan and Deci, 2001), with many of our activities revolving around the pursuit of a sense of purpose (Frankl, 1971; Bruner, 1990; Baumeister, 1991; Emmons, 1999; Peterson, 1999; Steger, 2009; Park, 2010; Markman et al., 2013). As one of the primary domains of human action, the workplace is increasingly being studied as a source of potentially meaningful experiences (Pratt and Ashforth, 2003; Rosso et al., 2010). Meaningful work experiences have a broad importance because they can help people to feel that they have a more meaningful life in general (Steger and Dik, 2009). All things being equal, people should accordingly be motivated to choose jobs that are perceived as meaningful, providing them with a sense of purpose and significance.

Despite this line of thinking, much of the research on job choice has focused on the importance of financial compensation, rather than the extent to which a job is considered to be personally meaningful (Chapman et al., 2005). Concerns about meaning should nonetheless play an important role in job choice decisions, especially given that pay level is only weakly correlated with job satisfaction (Judge et al., 2010). Because meaningful work can provide psychological benefits, it is possible that it will also decrease the importance of financial compensation when evaluating job opportunities. According to social exchange theory (Cropanzano and Mitchell, 2005), evaluations of employee-employer relationships involve a comparison of one’s own contributions (e.g., time and effort invested) with the benefits that are provided by employment. Importantly, the benefits that an employee derives from his or her work can involve a mixture of material and psychological reward (Foa and Foa, 1980). In principle, any decreases in one of these reward domains could be offset by increases in another without disrupting the overall perception of job-related reward. Because the opportunity to engage in meaningful work can provide a variety of psychological rewards, it should accordingly help to offset the need for material rewards. The current set of studies is designed to test this possibility, examining whether, and to what extent, people are willing to accept lower salaries in order to work in more meaningful jobs.

## Study 1

### Methods

The study’s procedure was approved by the Office of Research Ethics at the University of Toronto. Amazon’s Mechanical Turk (Mason and Suri, 2011; Landers and Behrend, 2015) was used to recruit 245 participants from the United States. *A priori* power analysis revealed that this sample size would have 95% power to detect the average social psychological effect size of *r* = 0.21 (Richard et al., 2003). Participants included 96 women and 149 men, with a mean age of 34.0 years (*SD* = 12.36) and a median yearly household income of $50,000 to $59,999 (range = less than $20,000 to greater than $150,000). This income distribution is consistent with the 2015 national median household income of $55,775. Approximately half of the sample (*n* = 130) currently worked in a full-time job (median yearly salary = $37,000). Participants were primarily Caucasian (82.9%), with smaller numbers of Asian (5.7%), Hispanic (5.7%), and African American (4.9%) respondents. The majority of the sample had 4-year college degrees (40%), 2-year college degrees (12.2%), or had not yet completed college (30.6%). An additional 9% of the sample had only a high school education and 8.2% had a graduate degree.

After providing their informed consent, participants were asked “What is a job or career that you are capable of doing that you think would provide you with a sense of personal meaning?” and “What is a job or career that you are capable of doing that you think would fail to provide you with a sense of personal meaning?” A total of 86 different meaningful jobs were listed, the most common of which were Teacher (26), Writer (19), Artist (13), Nurse (8), and working for a non-profit organization (8). In contrast, 64 different meaningless jobs were listed, including the most common entries of Accountant (22), Food Service Worker (16), Banker (15), Salesperson (14), and Office Worker (14). Notably, 44% of the jobs that were listed as being meaningful by one participant were listed by at least one other participant as lacking meaning. Similarly, 55% of the jobs that were listed as meaningless by one participant were listed as meaningful by someone else. This substantial overlap between the two categories indicates that the extent to which a job is considered meaningful is largely a subjective judgment. Additionally, 51% of the jobs that were listed as being meaningful were mentioned by only one participant, further suggesting that there is a large amount of individual variability when identifying meaningful positions.

After listing a meaningful and meaningless job, participants were asked “If you didn’t currently have a job, what is the lowest yearly salary (before tax) that you would realistically be willing to work for in the following job” and were presented with each of the two jobs that they had previously listed. A person’s minimum acceptable salary for a given job is a valid indicator of real-world salary evaluations because it serves as a key reference standard against which actual pay levels are compared (Rice et al., 1990). The extent to which an individual is satisfied with a given pay level thus depends to a large extent on how it compares to this minimum reference point (Locke, 1969).

Finally, participants completed a demographic questionnaire and were reimbursed for their time.

### Results

**FIGURE 1:**
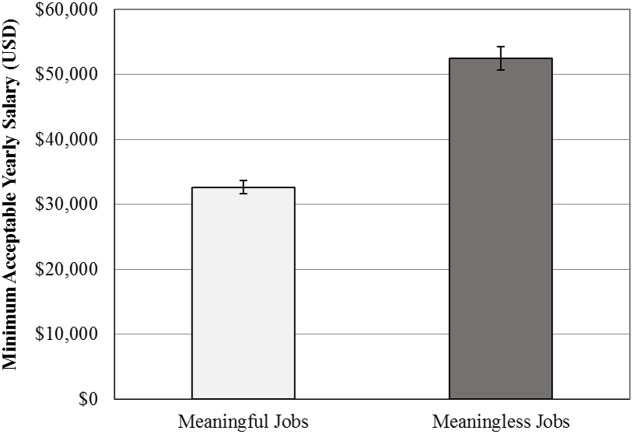
Average minimum acceptable salaries for meaningful and meaningless jobs in Study 1.

To test the hypothesis that people would accept lower minimum salaries in order to engage in more meaningful work, we compared participants’ minimum salaries across jobs. For meaningful jobs, participants were willing to accept an average minimum salary of $32,666 (*SD* = $15,060). For meaningless jobs, the minimum acceptable salary averaged at $52,498 (*SD* = $28,470). A paired-samples *t*-test confirmed our hypothesis and found that these two values were significantly different from one another, *t*(244) = 14.31, *p* < 0.001, *d* = 1.83 (see **Figure 1**). A bootstrap analysis with 5,000 resamples revealed the 95% confidence interval for this difference to range from $17,300 to $22,639, reflecting the average amount of yearly income that participants were willing to forfeit in order to work in a more rather than less meaningful job.

Given the large variability in minimally acceptable income levels across participants and jobs, the magnitude of the difference between meaningless and meaningful job salaries was divided by the minimum salary each participant required for a meaningless job. The resulting variable therefore reflects the percentage reduction in yearly income that each participant is willing to accept in order to work in the more meaningful position. A one-sample *t*-test confirmed that participants were willing to reduce their salaries by a significant percentage for the sake of having a meaningful job (*M* = 31.83%, *SD* = 23.71%), *t*(244) = 21.01, *p* < 0.001, Cohen’s *d* = 1.34.

We next examined whether any demographic characteristics might relate to the tradeoff between money and meaningful work. The percentage of their yearly incomes that participants were willing to forgo in order to have a more meaningful job showed no significant relationship with gender (*r* = -0.01, *p* = 0.93), age (*r* = -0.11, *p* = 0.10), marital status (*r* = -0.10, *p* = 0.12), yearly household income (*r* = 0.04, *p* = 0.57), or current employment status (*r* = 0.09, *p* = 0.17). There was a significant positive correlation with education level, however, such that greater education was associated with a willingness to accept larger salary reductions for personally meaningful work (*r* = 0.13, *p* = 0.04). Participants with children, on the other hand, reported a smaller minimum salary reduction for meaningful work compared to those who did not have children (*r* = -0.13, *p* = 0.04). Nonetheless, even participants with children (*n* = 92) were on average willing to accept a significantly lower salary in order to work in the more meaningful job (*M* = 27.8%, *SD* = 21.6%), *t*(91) = 12.37, *p* < 0.001, Cohen’s *d* = 1.29.

### Discussion

On average, participants reported minimum acceptable salaries that were 32% lower for personally meaningful jobs compared to jobs that were perceived as personally meaningless. This finding is particularly noteworthy when considering that nearly half of the jobs that were described as “meaningful” by at least one participant were also described as “meaningless” by others. What this suggests is that although the perception of meaningful work is a largely subjective appraisal, it is nonetheless associated with substantial value in the eyes of employees.

The willingness to accept lower salaries in exchange for meaningful work also emerged across a broad variety of job categories and income levels. Perhaps surprisingly, socioeconomic status, as quantified by yearly household income, showed no relationship with the monetary value of meaningful work. In particular, participants from lower income households were willing to accept a similar percentage reduction in their yearly salary in exchange for more personally meaningful jobs. Those participants who lacked a full-time job, and may as a result have been more motivated to find paid work, likewise did not report any less willingness to accept reduced salaries in exchange for meaningful positions. One possible explanation for this result is that the analyses were based on the percentage salary reduction for meaningful compared to meaningless work. Participants with lower household income levels reported lower minimum acceptable salary levels for both meaningful (*r* = 0.29, *p* < 0.01) and meaningless (*r* = 0.28, *p* < 0.01) jobs. An equivalent percentage reduction in salary thus corresponds to a lower absolute salary reduction for lower income participants. Indeed, the absolute difference between acceptable salaries for meaningful and meaningless jobs was larger for higher income earners (*r* = 0.17, *p* < 0.01).

Among demographic characteristics, only higher education levels predicted a willingness to give up an increased percentage of one’s salary in exchange for meaning, perhaps reflecting the educational emphasis on fostering a sense of personal autonomy (Deci et al., 1991). Conversely, participants who had at least one child were somewhat less likely to accept lower salaries for more meaningful work, perhaps because providing financial resources to one’s children can already be an important source of meaning (Christiansen and Palkovitz, 2001). It is also possible that parents need more money to raise their children, which in turn makes them somewhat less willing to sacrifice money for meaningful work. Nonetheless, parents were still willing to accept salaries that were an average of 28% lower for meaningful compared to meaningless positions.

Although this study highlights the willingness to accept lower salaries in exchange for personally meaningful work, it also has some limitations. Most importantly, the study relied on participants’ self-reported minimum acceptable salary levels for different jobs. It is certainly possible that when faced with actual job offers, participants would be willing to accept salaries that are lower than the reported values, or even refuse to accept offers with larger salaries. The current data nonetheless provide a useful estimate of real-world choices, as participant reports of minimum acceptable salary levels have a strong relation to their actual salary evaluations (Locke, 1969; Rice et al., 1990). Salaries thus tend to be viewed more or less positively depending on how they relate to one’s minimum acceptable salary as a reference point (cf. Kahneman and Tversky, 1979). Pay satisfaction can accordingly be predicted much more effectively when the discrepancy with one’s minimum acceptable salary is taken into account (Rice et al., 1990). Behavioral intentions in this domain are also likely to have a relatively high correlation with actual choices due to the fact that accepting a job offer is a major life decision that requires a great deal of deliberation (Sheeran, 2002).

A second limitation of the study is that participants were asked to think about jobs that clearly do or do not provide a sense of personal meaning. It is likely the case, however, that many jobs would not fit clearly into either of these categories. A job that provides a moderate amount of personal meaning might accordingly have a minimum acceptable salary level that is somewhere between the amounts reported for clearly meaningful and meaningless jobs. It is likely appropriate, therefore, to think of the 32% salary reduction as the upper limit of what people are generally willing to give up for meaningful work, reflecting the contrast between the most and least meaningful positions that they are realistically capable of doing.

Finally, it is also possible that participant responses were influenced by their beliefs about job-appropriate salaries. In particular, it could be that the jobs that people listed as meaningful are known to pay less overall, thereby leading participants to adopt lower minimum acceptable salaries based on realistic expectations. While job-specific anchoring of salary expectations cannot be ruled out completely, it should be noted that the average salaries listed on O^∗^Net are actually higher for the top five meaningful jobs ($55,486) compared to the top five meaningless jobs ($44,522). In other words, the more meaningful jobs don’t necessarily have lower salaries than the meaningless ones, especially given the large amount of overlap between the two lists.

## Study 2

Because participants in Study 1 were free to list their own examples of meaningful and meaningless jobs, there may have been extraneous factors that influenced the acceptable salary levels for each. In particular, any differences in acceptable salaries may have been due to the different job characteristics of the positions being compared, rather than differences in meaningfulness alone. Although job characteristics are known to influence the extent to which work is perceived as meaningful (Hackman and Oldham, 1980; Johns et al., 1992), a cleaner test of the hypothesis that the subjective meaning of work has an influence on acceptable salary levels requires that all job characteristics are held constant. Study 2 was designed to address this concern by holding the type of job constant across participants, while allowing the apparent meaningfulness of the position to vary. Study 2 also employs a between-subject design to eliminate the concern that the comparisons between meaningful and meaningless jobs in Study 1 may have suffered from demand characteristics, thereby exaggerating any differences in the minimum acceptable salaries.

### Methods

Holding the type of job constant across participants required that we first identify a job that would be commonly recognized by most respondents. It was also important to use a variety of jobs to examine whether the effects of subjective meaning might be job-specific, or depend upon the average salary level of the position. Occupational data from the United States was used to identify common jobs in the upper, middle, and lower thirds of the average salary distribution. The jobs that were chosen include lawyer (median salary = $115,820; workers = 779,000), elementary school teacher (median salary = $54,890; workers = 1,358,000), and delivery services driver (median salary = $29,850; workers = 885,000).

Amazon’s Mechanical Turk was used to recruit 303 participants from the United States. Participants included 175 women and 128 men, with a mean age of 34.0 years (*SD* = 12.36) and a median yearly household income of $50,000 to $59,999 (range = less than $20,000 to greater than $150,000), which is again consistent with the national average. The majority of the sample (*n* = 187) currently worked in a full-time job (median yearly salary = $40,000). Participants were primarily Caucasian (76.2%), with smaller numbers of African American (7.9%) Asian (7.0%), and Hispanic (4.3%) respondents. The majority of the sample had 4-year college degrees (36.3%), 2-year college degrees (11.6%), or had not yet completed college (27.4%). An additional 8.6% of the sample had only a high school education and 15.8% had a graduate degree.

After providing their informed consent, participants were randomly presented with one of six brief job descriptions in a 3 (job type: lawyer, elementary school teacher, delivery services driver) × 2 (condition: meaningful, control) between-subject design. Job descriptions for each position were taken directly from the Occupational Information Network (Peterson et al., 2001). Participants in the “meaningful” condition were additionally asked to “Please think about, and write down, how working as a [JOB TITLE] might provide you with a sense of personal meaning. Even if it isn’t obvious, try to imagine how working in this job might give you a sense of purpose and meaningful work.” These instructions were designed to evoke the perception of subjective meaning without altering the specific content of the job descriptions. All participants were then asked “If you didn’t currently have a job, what is the lowest yearly salary (before tax in US$) that you would realistically be willing to work for in this job?”

It was hypothesized that participants would report lower acceptable salary levels when they had first reflected on how meaningful the potential job could be.

### Results

A review of the answers provided in the meaning reflection condition confirmed that all participants took the instructions seriously and provided thoughtful responses (e.g., “Delivering packages would allow me to put a smile on people’s faces, which would bring meaning to my life” and “Teaching young children is very rewarding. You are responsible for shaping the minds of youngsters in a way that forever changes them”).

**FIGURE 2:**
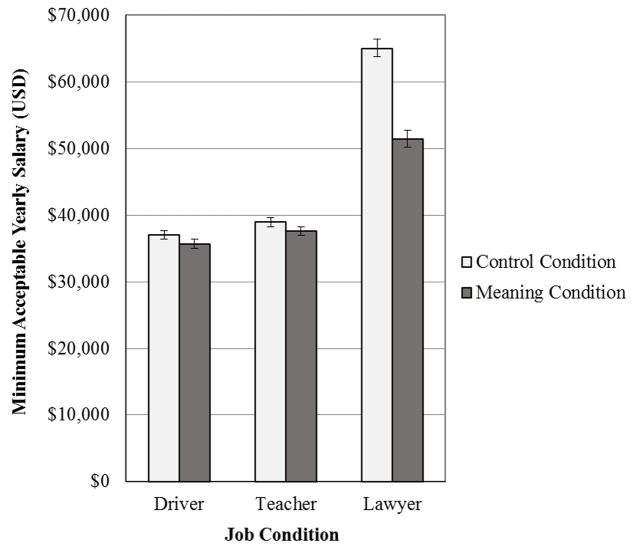
Average minimum acceptable salaries for each of the job and meaning conditions in Study 2.

A 3 × 2 factorial ANOVA was used to test the effects of job type and the personal meaning reflection on minimum acceptable salary levels. A significant main effect was observed for job type, *F*(2,285) = 54.16, *p* < 0.01, indicating different salary preferences in order to work as a lawyer (*M* = $58,667, *SD* = $23,711), elementary school teacher (*M* = $38,363, *SD* = $11,419), or delivery services driver (*M* = $36,425, *SD* = $11,498). As hypothesized, a main effect of meaningfulness also emerged, *F*(1,285) = 8.15, *p* < 0.01, with participants in the meaning reflection condition reporting lower minimum salaries (*M* = $41,670, *SD* = $17,244) than those in the control condition (*M* = $46,657, *SD* = $20,650). A significant interaction effect also emerged, *F*(2,285) = 4.60, *p* = 0.01, indicating that the effect of the meaning reflection varied by job type. Simple main effects analysis revealed that the meaning reflection had a significant impact in the lawyer condition, *F*(1,285) = 16.96, *p* < 0.01, but not for those in the elementary school teacher, *F*(1,285) = 0.17, *p* = 0.69, or delivery services driver conditions, *F*(1,285) = 0.17, *p* = 0.68. Among those reading the job description for lawyers, minimum salary levels were lower in the meaning reflection condition (*M* = $51,444, *SD* = $21,706) compared to the control condition (*M* = $65,039, *SD* = $23,776). **Figure 2** presents these results graphically. The same pattern of results was obtained when including age, gender, employment status, education level, marital status, parental status, and yearly household income as covariates.

### Discussion

Study 2 experimentally manipulated the apparent meaningfulness of three jobs with different income levels. Consistent with Study 1, participants reported significantly lower minimum acceptable salaries for working as a lawyer when they had first been asked to reflect on how the job could be personally meaningful to them. No such differences were found with the other two job types, however, suggesting that the effects might have some degree of job-specificity. Given that lawyers also had the highest salary of the three jobs that were used in the study, it may be the case that people are more likely to accept lower salaries for meaningful work once their base incomes have passed some minimum threshold. This would be consistent with the finding that the positive relationship between income and well-being tapers off once a person’s basic needs are met (Diener and Biswas-Diener, 2002). An alternative possibility is that the job specificity of the experimental results can be explained by the different stereotypes that are held about each of the jobs. People may already view the teacher role as being inherently meaningful, for example, because of the social impact associated with it. Asking people to further reflect on how meaningful it would be to work as a teacher thus wouldn’t have a large influence on baseline perceptions of meaningfulness. Lawyers, on the other hand, tend to have a more negative public image, which could result in lower baseline perceptions of occupational meaningfulness and an increased effectiveness of the meaning reflection. This explanation would not, however, explain the non-significant effect when considering a job as a delivery services driver (which also lacks an obvious inherent meaningfulness).

## Study 3

Studies 1 and 2 both relied on participants’ self-report of their minimum acceptable salary levels when rating jobs with different levels of personal meaningfulness. Although these ratings provide a useful proxy for real-world decisions, they are not perfect indicators of an individual’s actual job choices. In particular, the decision to accept lower salaries for meaningful work may be different when thinking about one’s actual job options. To address this limitation, Study 3 examines the tradeoff between money and meaning as it relates to decisions about currently held jobs in a large cross-cultural sample of full-time workers. It was hypothesized that those employees who find their jobs to be more meaningful would also be less interested in working for higher pay in a different position.

### Methods

Data for Study 3 were obtained from the International Social Survey Program’s Work Orientation Module. The survey was administered at two time points, once in 2005 and again in 2015. The 2005 survey asked 43,441 participants from 31 different (primarily developed) countries to answer a variety of questions about their work lives. The 2015 survey asked similar questions to a second group of 33,105 participants from 24 countries (see International Social Survey Programme [ISSP], 2017 for the full details about the panel data and its administration). Because we were interested in real-world job attitudes, we focused our analyses on the participants who were currently employed in full-time jobs. In the 2005 survey, the full-time employee subsample had 18,919 participants, which included 9,941 men and 8,970 women (eight participants unspecified), with a mean age of 41.7 years (*SD* = 12.75). Participants had an average of 12.70 years of education (*SD* = 3.79) and worked in 529 distinct job categories. In the 2015 survey, the full-time employee subsample had 18,472 participants, which included 9,346 men and 9,126 women (five participants unspecified), with a mean age of 43.41 years (*SD* = 13.06). Participants had an average of 13.53 years of education (*SD* = 4.10) and worked in 561 distinct job categories.

Although no direct measure of meaningful work was included in the survey, a composite measure was created by averaging responses to three different questions: “My job is interesting,” “In my job I can help other people,” and “My job is useful to society” (*M_2005_* = 2.21, *SD_2005_* = 0.85, *α_2005_* = 0.66; *M_2015_* = 2.07, *SD_2015_* = 0.77, *α_2015_* = 0.71). Each of these items was rated on a Likert scale ranging from 1 (Strongly Agree) to 5 (Strongly Disagree). These items are considered to be good proxies for meaningful work because the amount of intrinsic interest and social value associated with a job are positively correlated with the experience of meaningfulness (Grant, 2007; Rosso et al., 2010). The composite variable was then used to predict participant responses to the question “I would turn down another job that offered quite a bit more pay in order to stay with this organization” (*M_2005_* = 3.51, *SD_2005_* = 1.67; *M_2015_* = 3.16, *SD_2015_* = 1.23), which was rated on a Likert scale ranging from 1 (Strongly Agree) to 5 (Strongly Disagree).

### Results

Data from the two assessment periods were pooled together for the analysis, with survey year coded as 0 (2005) or 1 (2015). Participants’ willingness to turn down a higher-paying job with another organization was regressed upon the composite measure of meaningful work in a cross-classified hierarchical linear model. Random intercepts were included for the participants’ country of origin and job category as identified by the International Standard Classification of Occupations (Elias, 1997). The meaningful work variable was jointly centered within country, job category, and data collection year, thereby eliminating any between-group variance associated with these factors. The results accordingly reflect the average association between meaningful work and the willingness to turn down higher-paying jobs within each country, job category, and time point. In order to examine whether this relationship has changed over time, the model also included a random slope for the meaningful work variable, a fixed effect for year of data collection, and the interaction between meaningful work and survey year.

**FIGURE 3:**
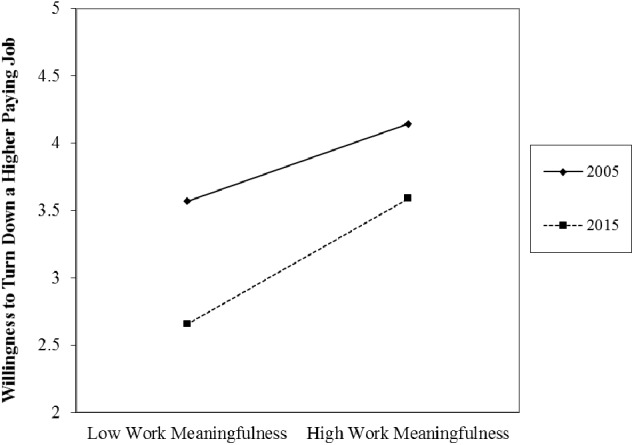
The relationship between work meaningfulness and the willingness to turn down a higher paying job in 2005 and 2015.

Among participants of the 2005 survey, the extent to which a person reported meaningful work experiences positively predicted his or her willingness to turn down a higher-paying job with another organization (*b* = 0.377, *SE* = 0.021, *t* = 18.20, *p* < 0.001). A significant interaction with the year of data collection indicated that this relationship became even stronger over time (*b* = 0.090, *SE* = 0.028, *t* = 3.16, *p* < 0.01). A significant main effect of survey year also emerged, indicating that people became more willing to leave their organizations for higher paying opportunities in 2015 compared to 2005 (*b* = -0.366, *SE* = 0.023, *t* = -16.17, *p* < 0.001). See **Figure 3** for a graphical comparison of the two time points.

Covariance parameters indicated that the relationship between meaningful work and the willingness to turn down a higher-paying job did not vary significantly by country (Variance Estimate = 0.004, Wald *Z* = 1.41, *p* = 0.16) or job type (Variance Estimate = 0.008, Wald *Z* = 1.72, *p* = 0.09), although the overall likelihood of turning down a higher-paying job did vary significantly by country (Variance Estimate = 0.066, Wald *Z* = 4.17, *p* < 0.001) and job type (Variance Estimate = 0.035, Wald *Z* = 7.44, *p* < 0.001). Removing the random slope for the meaningful work variable did not alter the main finding, nor did including the effects of age, gender, or income as covariates.

### Discussion

In Study 3, two sets of large cross-national panel data collected in 2005 and 2015 were used to gauge the relationship between the experience of meaningful work and the willingness to turn down higher paying job opportunities. Consistent with the first two studies, higher levels of work meaningfulness were associated with a greater willingness to forego higher paying job options. Furthermore, this relationship was stronger among employees in 2015 compared to 2005, perhaps reflecting a greater emphasis on meaningful work among the younger cohort of employees (Deal et al., 2010). This change is a striking contrast against the overall decrease in employees’ willingness to reject higher paying job offers, a finding that is consistent with increased job and organizational mobility among younger generations (Lyons et al., 2015).

It is also interesting to note that the relationship between meaningful work and the willingness to turn down a higher paying job did not vary significantly by country or job category, although the general willingness to turn down higher paying opportunities did vary with both of these variables. This suggests that people are willing to accept lower salaries for more meaningful work across a diversity of cultural and occupational contexts. A caveat to this point, however, is that data were collected primarily from developed nations. It is thus still possible that this effect might vary in other national contexts, or in jobs that weren’t sampled in the present dataset. In the current data, however, the effect appears to be robust across countries and job categories.

## Study 4

Although Study 3 provides strong support for the general willingness to accept lower salaries for more meaningful work, it fails to make an important distinction that has emerged in the meaning literature. In particular, organizational scholars have distinguished between meaning *in* work and meaning *at* work (Pratt and Ashforth, 2003). Meaning *in* work refers to the extent to which an individual’s work activities themselves are perceived as meaningful (i.e., the person has a meaningful *job*). Conversely, meaning *at* work refers to the extent to which employees perceive their involvement within an organizational community as meaningful (regardless of the particular job that is being performed). The phrasing of the dependent variable in the panel data used for Study 3 referenced both of these types of meaning, because it mentioned a “job” and an “organization.” As a result, it is not clear whether, and to what extent, the findings relate to having meaning in work or meaning at work. Study 4 was designed to more clearly differentiate these options. It was hypothesized that those employees who have more meaningful work experiences would be less interested in working for higher pay (1) in a different job within the same organization, and (2) in the same job within a different organization.

### Methods

Amazon’s Mechanical Turk was used to recruit 441 participants from the United States who are currently working in a full-time job. Among the 441 participants, 2 indicated that they did not answer all questions honestly and 9 of them failed the attention check question asking them to select a particular response option. Once these 11 participants were removed, the final sample consisted of 430 participants, including 185 women, 240 men, 1 other, and 4 missing answers. Participants had a mean age of 35.78 years (*SD* = 10.80) and a median yearly household income of $50,000 to $59,999 (range = less than $20,000 to greater than $150,000). Participants were primarily Caucasian (74.00%), with smaller numbers of Asian (9.60%), African American (7.30%), and Hispanic (6.30%) respondents. Most of the sample had 4-year college degrees (44.60%), some college but no degrees (18.80%), or 2-year college (11.70%). The occupations of the participants were diverse, containing jobs from 34 different occupational categories.

Participants were given access to the questionnaires if they indicated that they had a full-time job. Upon beginning the study, participants were asked to rate their agreement with three statements on a seven-point Likert scale ranging from Strongly Disagree to Strongly Agree. The first question focused on meaning *in* work by asking about the job specifically: “I would turn down a higher-paying job with my current employer in order to keep working in the job I am in now” (*M* = 2.90, *SD* = 1.66). The second question focused on meaning at work by asking about the organization: “I would turn down a higher-paying offer for the same job, but from a different company, in order to stay with my current employer” (*M* = 3.38, *SD* = 1.78). Finally, we also included the same question from Study 3, which includes a mixture of these two aspects of meaningful work: “I would turn down another job that offered quite a bit more pay in order to stay with this organization” (*M* = 3.07, *SD* = 1.79).

After answering these questions, participants’ experiences of meaningful work were assessed with the work and meaning inventory (WAMI, Steger et al., 2012). The WAMI includes 10 items assessing the extent to which people find their work to be personally meaningful (e.g., “I have discovered work that has a satisfying purpose”). Participants indicated their agreement to these items on a five-point scale ranging from Strongly Disagree to Strongly Agree (*M* = 3.36, *SD* = 0.98; α = 0.94). Participants then completed a demographic questionnaire where they reported their age, gender, ethnicity, annual income, and education.

### Results

In a replication of the Study 3 results, participants who experienced more meaningful work lives were significantly more likely to turn down another job with higher pay in order to stay with their current organization (*r* = 0.34, *p* < 0.001). Participants reporting more meaningful work experiences were also more likely to turn down a different but higher paying job in the same organization (*r* = 0.29, *p* < 0.001), and turn down the same job but for higher pay in a different organization (*r* = 0.38, *p* < 0.001). These correlations remained significant when statistically controlling for demographic information.

A regression analysis was conducted to examine the contributions of job-specific and organization-specific meaning to the general question that was posed in the Study 3 panel data. The willingness to turn down another job at a different organization was simultaneously predicted by the willingness to turn down the same job within a different organization (β = 0.59, *t* = 15.15, *p* < 0.001) and the willingness to turn down a different job within the same organization (β = 0.26, *t* = 6.69, *p* < 0.001). Collectively, these two variables accounted for 59% of the variance in the general panel data question.

### Discussion

In a sample of full-time employees spanning many different job types, those who experienced their current jobs as relatively more meaningful were less likely to accept another job for more pay at a different organization. These effects emerged when looking specifically at turning down higher pay for the same job at a different organization and turning down higher pay for a different job with one’s current employer. The willingness to accept a lower salary for more meaningful work thus appears to apply both to meaning in work (relating to the job itself) and meaning at work (relating to the organizational context within which work is performed).

## General Discussion

Financial concerns have a strong influence on job choice behavior (Chapman et al., 2005). Nonetheless, the current results indicate that people are willing to accept significantly lower salaries in exchange for more meaningful work. Study 1 demonstrated this effect by asking participants to compare their own self-generated examples of meaningful and meaningless jobs. Study 2 showed the effect with an experimental manipulation of meaning across three pre-defined job categories. Study 3 revealed a similar pattern in a large international sample in which people reflected on their current jobs. Study 4 replicated this finding when asking specifically about higher-paying jobs and higher-paying organizations. Collectively, these studies provide evidence that perceptions of meaningful work are able to reduce the emphasis on financial concerns when making job choices. These findings are consistent with theoretical frameworks that emphasize the subjective value of personally meaningful activities (Frankl, 1971; Bruner, 1990; Baumeister, 1991; Emmons, 1999; Peterson, 1999; Markman et al., 2013). Within the framework of Social Exchange Theory, meaningful work provides employees with a number of psychological rewards that can offset the need for financial compensation (Cropanzano and Mitchell, 2005).

An interesting implication of these studies is that companies may be able to save costs by hiring employees who find the work to be more personally meaningful. In contrast, the current data suggest that those employees who don’t find their work to be meaningful will expect higher levels of financial compensation for a given job. Because perceptions of meaning are inherently subjective, they may be a useful target for managerial efforts to contain costs—especially in organizations with limited financial resources. On the other hand, if companies purposefully try to exploit their employees’ inherent sense of work meaningfulness, undesirable outcomes might emerge. For example, purposeful low payment may lead to a perception of unfairness among employees, which could disrupt organizational commitment (Meyer et al., 2002). Encouraging employees to make too many self-sacrifices for the sake of meaningful work may similarly produce negative outcomes by disrupting their comfort, health, or work-life balance (Dempsey and Sanders, 2010; Berkelaar and Buzzanell, 2015). Companies should therefore be careful when applying these results to avoid any undesired outcomes.

An important question about the current results is the extent to which they are influenced by person or job-specific factors. Study 2 suggests that people may be more likely to accept lower salaries for meaningful work when they are already above a certain income level (i.e., the effect was only observed for lawyers, but not for teachers or delivery truck drivers). This finding did not emerge in Study 1, however, which found no relationship between participant income levels and the percentage of their salary that they are willing to give up for meaningful work. Studies 3 and 4 likewise found that the willingness to turn down higher paying jobs in order to remain in a meaningful position was observed across a wide variety of jobs and income levels.

Another possibility is that the results apply mainly to highly educated individuals. Indeed, the online samples from Studies 1, 2, and 4 were more highly educated than the national average in the United States. In all of these studies, however, the effects remained significant when controlling for education and other demographic variables. Even in Study 1, where a significant interaction with education emerged, the effect was still present (in slightly attenuated form) among those with less education. Study 3 similarly demonstrated the same effect when using nationally representative samples of employees from many different countries. The overall effect thus appears to be generalizable to a broad population, but it may still be useful for future research to examine the potential influence of job, person, or culture-specific factors on the tradeoff between money and meaningfulness.

Researchers are increasingly interested in the many individual and organizational benefits that accompany the perception that one’s work is personally meaningful (Ryan and Deci, 2001; Pratt and Ashforth, 2003; Rosso et al., 2010; Steger et al., 2012). Despite the tendency to focus on financial reward as one of the most important aspects of a job, the current research shows that people are generally willing to forgo larger salaries in the pursuit of more meaningful work. Across a wide variety of job categories, countries, and income levels, the desire for a meaningful job is able to offset purely financial concerns.

## Ethics Statement

This study was carried out in accordance with the recommendations of the American Psychological Association’s Ethics Code. All participants gave written informed consent in accordance with the Declaration of Helsinki.

## Author Contributions

All authors listed have made a substantial, direct, and intellectual contribution to the work, and approved it for publication.

## Conflict of Interest Statement

The authors declare that the research was conducted in the absence of any commercial or financial relationships that could be construed as a potential conflict of interest. The reviewer TS and handling Editor declared their shared affiliation.

## References

[B1] Barrick M. R. Mount M. K. Li N. (2013). The theory of purposeful work behavior: the role of personality, higher-order goals, and job characteristics. *Acad. Manag. Rev.* 38 132–153. 10.5465/amr.2010.0479

[B2] Baumeister R. F. (1991). *Meanings of Life.* New York, NY: Guilford Press.

[B3] Berkelaar B. L. Buzzanell P. M. (2015). Bait and switch or double-edged sword? The (sometimes) failed promises of calling. *Hum. Relat.* 68 157–178. 10.1177/0018726714526265

[B4] Bruner J. (1990). *Acts of Meaning.* Cambridge, MA: Harvard University Press.

[B5] Bunderson J. S. Thompson J. A. (2009). The call of the wild: zookeepers, callings, and the double-edged sword of deeply meaningful work. *Adm. Sci. Q.* 54 32–57. 10.2189/asqu.2009.54.1.32

[B6] Chapman D. S. Uggerslev K. L. Carroll S. A. Piasentin K. A. Jones D. A. (2005). Applicant attraction to organizations and job choice: a meta-analytic review of the correlates of recruiting outcomes. *J. Appl. Psychol.* 90 928–944. 10.1037/0021-9010.90.5.928 16162065

[B7] Christiansen S. L. Palkovitz R. (2001). Why the ‘good provider’ role still matters: providing as a form of paternal involvement. *J. Fam. Issues* 22 84–106. 10.1177/019251301022001004

[B8] Cropanzano R. Mitchell M. S. (2005). Social exchange theory: an interdisciplinary review. *J. Manage.* 31 874–900. 10.1177/0149206305279602

[B9] Deal J. J. Altman D. G. Rogelberg S. G. (2010). Millennials at work: what we know and what we need to do (if anything). *J. Bus. Psychol.* 25 191–199. 10.1007/s10869-010-9177-2

[B10] Deci E. L. Vallerand R. J. Pelletier L. G. Ryan R. M. (1991). Motivation and education: the self-determination perspective. *Educ. Psychol.* 26 325–346. 10.1080/00461520.1991.9653137

[B11] Dempsey S. E. Sanders M. L. (2010). Meaningful work? Nonprofit marketization and work/ life imbalance in popular autobiographies of social entrepreneurship. *Organization* 17 437–459. 10.1177/1350508410364198

[B12] Diener E. Biswas-Diener R. (2002). Will money increase subjective well-being? *Soc. Indic. Res.* 57 119–169. 10.1023/A:1014411319119

[B13] Elias P. (1997). *Occupational Classification (ISCO-88): Concepts, Methods, Reliability, Validity and Cross-National Comparability.* Available at: https://ideas.repec.org/p/oec/elsaaa/20-en.html 10.1787/304441717388

[B14] Emmons R. A. (1999). *The Psychology of Ultimate Concerns: Motivation and Spirituality in Personality.* New York, NY: Guilford Press.

[B15] Foa E. B. Foa U. G. (1980). “Resource Theory,” in *Social Exchange* eds Gergen K. J. Greenberg M. S. Willis R. H. (New York, NY: Springer) 77–94.

[B16] Frankl V. (1971). *Man’s Search for Meaning.* New York, NY: Pocket Books.

[B17] Grant A. M. (2007). Relational job design and the motivation to make a prosocial difference. *Acad. Manage. Rev.* 32 393–417. 10.5465/AMR.2007.24351328

[B18] Grant A. M. (2008). The significance of task significance: job performance effects, relational mechanisms, and boundary conditions. *J. Appl. Psychol.* 93 108–124. 10.1037/0021-9010.93.1.108 18211139

[B19] Greenhaus J. H. Bedeian A. G. Mossholder K. W. (1987). Work experiences, job performance, and feelings of personal and family well-being. *J. Vocat. Behav.* 31 200–215. 10.1016/0001-8791(87)90057-1

[B20] Hackman J. R. Oldham G. R. (1976). Motivation through the design of work: test of a theory. *Organ. Behav. Hum. Perform.* 16 250–279. 10.1016/0030-5073(76)90016-7

[B21] Hackman J. R. Oldham G. R. (1980). *Work Redesign.* Reading, MA: Addison-Wesley.

[B22] International Social Survey Programme [ISSP] (2017). *Work Orientations Module.* Available at: https://www.gesis.org/issp/modules/issp-modules-by-topic/work-orientations/

[B23] Johns G. Xie J. L. Fang Y. (1992). Mediating and moderating effects in job design. *J. Manage.* 18 657–676. 10.1177/014920639201800404

[B24] Judge T. A. Piccolo R. F. Podsakoff N. P. Shaw J. C. Rich B. L. (2010). The relationship between pay and job satisfaction: a meta-analysis of the literature. *J. Vocat. Behav.* 77 157–167. 10.1016/j.jvb.2010.04.002

[B25] Judge T. A. Watanabe S. (1993). Another look at the job satisfaction-life satisfaction relationship. *J. Appl. Psychol.* 78 939–948. 10.1037/0021-9010.78.6.939

[B26] Kahneman D. Tversky A. (1979). Prospect theory: an analysis of decision under risk. *Econometrica* 47 263–291. 10.1007/s11336-014-9425-x

[B27] Kanungo R. N. (1982). *Work Alienation: An Integrative Approach.* New York, NY: Praeger.

[B28] Landers R. N. Behrend T. S. (2015). An inconvenient truth: arbitrary distinctions between organizational, mechanical turk, and other convenience samples. *Ind. Organ. Psychol.* 8 142–164. 10.1017/iop.2015.13

[B29] Locke E. A. (1969). What is job satisfaction? *Organ. Behav. Hum. Perform.* 4 309–336. 10.1016/0030-5073(69)90013-0

[B30] Lyons S. T. Schweitzer L. Ng E. S. W. (2015). How have careers changed? An investigation of changing career patterns across four generations. *J. Manag. Psychol.* 30 8–21. 10.1108/JMP-07-2014-0210

[B31] Markman K. D. Proulx T. Lindberg M. J. (eds) (2013). *The Psychology of Meaning.* Washington, DC: American Psychological Association 10.1037/14040-000

[B32] Maslach C. Schaufeli W. B. Leiter M. P. (2001). Job burnout. *Annu. Rev. Psychol.* 52 397–422. 10.1146/annurev.psych.52.1.397 11148311

[B33] Mason W. Suri S. (2011). Conducting behavioral research on Amazon’s Mechanical Turk. *Behav. Res. Methods* 44 1–23. 10.3758/s13428-011-0124-6 21717266

[B34] May D. R. Gilson R. L. Harter L. M. (2004). The psychological conditions of meaningfulness, safety and availability and the engagement of the human spirit at work. *J. Occup. Organ. Psychol.* 77 11–37. 10.1348/096317904322915892

[B35] Meyer J. P. Stanley D. J. Herscovitch L. Topolnytsky L. (2002). Affective, continuance, and normative commitment to the organization: a meta-analysis of antecedents, correlates, and consequences. *J. Vocat. Behav.* 61 20–52. 10.1006/jvbe.2001.1842

[B36] Park C. L. (2010). Making sense of the meaning literature: an integrative review of meaning making and its effects on adjustment to stressful life events. *Psychol. Bull.* 136 257–301. 10.1037/a0018301 20192563

[B37] Peterson J. B. (1999). *Maps of Meaning: The Architecture of Belief.* New York, NY: Routledge.

[B38] Peterson N. G. Mumford M. D. Borman W. C. Jeanneret P. R. Fleishman E. A. Levin K. Y. (2001). Understanding work using the occupational information network (o^∗^net): implications for practice and research. *Pers. Psychol.* 54 451–492. 10.1111/j.1744-6570.2001.tb00100.x

[B39] Pratt M. G. Ashforth B. E. (2003). “Fostering meaningfulness in working and at work,” in *Positive Organizational Scholarship: Foundations of a New Discipline* eds Cameron K. S. Dutton J. E. Quinn R. E. (Oakland, CA: Berrett-Koehler) 309–327.

[B40] Rice R. W. Phillips S. M. McFarlin D. B. (1990). Multiple discrepancies and pay satisfaction. *J. Appl. Psychol.* 75 386–393. 10.1037/0021-9010.75.4.386

[B41] Richard F. D. Bond C. F. Stokes-Zoota J. J. (2003). One hundred years of social psychology quantitatively described. *Rev. Gen. Psychol.* 7 331–363. 10.1037/1089-2680.7.4.331

[B42] Rosso B. D. Dekas K. H. Wrzesniewski A. (2010). On the meaning of work: a theoretical integration and review. *Res. Organ. Behav.* 30 91–127. 10.1016/j.riob.2010.09.001

[B43] Ryan R. M. Deci E. L. (2001). On happiness and human potentials: a review of research on hedonic and eudaimonic well-being. *Annu. Rev. Psychol.* 52 141–166. 10.1146/annurev.psych.52.1.141 11148302

[B44] Ryff C. D. (1989). Happiness is everything, or is it? Explorations on the meaning of psychological well-being. *J. Pers. Soc. Psychol.* 57 1069–1081. 10.3109/09638288.2010.503835

[B45] Schnell T. Höge T. Pollet E. (2013). Predicting meaning in work: theory, data, implications. *J. Posit. Psychol.* 8 543–554. 10.1080/17439760.2013.830763

[B46] Seeman M. (1959). On the meaning of alienation. *Am. Sociol. Rev.* 24 783–791. 10.2307/2088565

[B47] Shantz A. Alfes K. Truss C. (2014). Alienation from work: marxist ideologies and twenty-first-century practice. *Int. J. Hum. Resour. Manage.* 25 2529–2550. 10.1080/09585192.2012.667431

[B48] Sheeran P. (2002). Intention—behavior relations: a conceptual and empirical review. *Eur. Rev. Soc. Psychol.* 12 1–36. 10.1080/14792772143000003

[B49] Steger M. F. (2009). “Meaning in life,” in *Oxford Handbook of Positive Psychology* 2nd Edn eds Lopez S. J. Snyder C. R. (New York, NY: Oxford University Press) 679–687.

[B50] Steger M. F. Dik B. J. (2009). If one is looking for meaning in life, does it help to find meaning in work? *Appl. Psychol. Health Well Being* 1 303–320. 10.1111/j.1758-0854.2009.01018.x

[B51] Steger M. F. Dik B. J. Duffy R. D. (2012). Measuring meaningful work: the work and meaning inventory (WAMI). *J. Career Assess.* 20 322–337. 10.1177/1069072711436160

[B52] Tait M. Padgett M. Y. Baldwin T. T. (1989). Job and life satisfaction: a reevaluation of the strength of the relationship and gender effects as a function of the date of the study. *J. Appl. Psychol.* 74 502–507. 10.1037/0021-9010.74.3.502

